# Cast Clasp Technic in Removable Bridgework

**Published:** 1920-04

**Authors:** Norman B. Nesbett

**Affiliations:** Boston, Mass.


### CAST CLASP TECHNIC IN REMOVABLE BRIDGE-WORK

BY NORMAN B. NESBETT, D.M.D., BOSTON, MASS.

Technic may be defined as the method by which an artist uses his materials to express his mental conceptions. Hence the higher those mental conceptions, the more carefully planned will be the technic elaborated to fulfill those conceptions. A technic planned with the sole idea of producing something must always fall before the technic of the one whose vision leads him to build a technic that turns vision into actual result.

Many examples to illustrate the foregoing might be given, but one will suffice. It has become an axiom that the perfection of product of the gold inlay worker is in direct ratio to the care he uses in following out the technic employed.

The cast clasp, used as an anchorage for removable bridge-work, has proved its worth, and used where indicated, *where mutilation of the abutment teeth is not desirable,* can not be excelled by any known attachment now before the dental profession.

The experimental work done to produce the technic first presented by me to the profession in 1915, extended over a two-year period of 1913 and 1914. During that two-year period of experimental work many ideas and methods were tried and finally discarded—this with the vision ever in mind of ultimately placing before the dental profession a completed technic, scientific and yet at the same time practical and simple as possible. Of the discarded methods of producing a cast clasp, I will mention the following:

*Experiment No. 1.* Adapting casting wax and other wax directly to the abutment teeth in the mouth and attempting to remove the pattern.

*Experiment No. 2.* Adapting base plate and other kinds of wax to the teeth on a plaster model and casting, first, by cutting the teeth off the model, investing the whole mass, and casting; secondly, by removing the wax pattern.

*Experiment No. 3.* Painting liquid casting wax on a model of the abutment tooth poured with inlay investment, investing the tooth and pattern and casting. This same method was also tried with variations, such as: first, adapting thin casting wax to the tooth and building up the pattern to the desired thickness with other casting wax; second, adapting a thin pure gold matrix and then building up with casting wax; third, using a harder refractory material than inlay investment and casting direct to the tooth. Experiment No. 3, with its variations, is the popular method followed by those who believe that the present technic is too difficult. If ease of execution had been the only desideratum, our experiments would have ended with Experiment No. 3.

Quite satisfactory cast clasps can be produced by the use of all the foregoing methods.

Experiments were then conducted on models prepared with removable teeth, Weinstein's artificial stone being first used. This, although far harder than plaster of paris and indispensable in practically all prosthetic operations, proved too soft and amalgam was then substituted. These amalgam teeth proved satisfactory and are used at present. Experiments were also tried with the use of horizontal notches cut into the enamel of the buccal and lingual surfaces of the clasped teeth, and with the use of short round-headed pins or spuds set into the buccal surfaces of the abutment teeth. These pins engaged corresponding depressions in the buccal arms of the clasps, much in the manner of a glove fastener. As the idea of these devices is to gain additional retention to compensate for a poorly fitting clasp, and as the cast clasp should be used with the idea of *avoiding* tooth mutilation, the writer considers their use contraindicated. Indeed, he considers their use in an average case a confession of inability to make a proper fitting cast clasp.

Early experiments showed that it was very difficult in all cases, and almost impossible in some cases, to test the proper fit of a cast clasp by trying it on the abutment tooth in the patient's mouth. Therefore some other means of obtaining the correct relations of the clasp to the teeth, other than the customary methods of first taking an impression with the clasps fitted to the patient's teeth, had to be devised.

Our technic was developed with the idea of having an unblemished model on which to build the case, take relations of clasps in their *correct* positions, and make final fittings and adjustments; but, best of all, it enables us to properly design our clasps, and know that we can get the bridge easily in and out of position when completed. The technician who destroys his model at any phase of the process is obliged to wait for a trial in the patient's mouth with the result that much tedious blind grinding often has to be done before the piece can be adjusted.

"The practice of every art," says Professor Huxley, "implies a certain knowledge of natural causes and effects; and the improvement of the art depends upon our learning more and more of the properties and powers of natural objects, and discovering how to turn the properties and powers of things and the connections of cause and effect among them to our own advantage." The most practical thing in the world is the most scientific. In fact, nothing becomes really practical until it is put on a scientific basis; that is, until its relation to other facts is known and noted. To be scientific as well as practical, a cast clasp should not be a mere inert, bulky ring of metal, but should be resilient to a slight degree; not much heavier than a wrought clasp of 24 gauge (B. & S. Standard), but most important of all, should grasp the tooth tighter at its ends than at anywhere else. This last-named property is obtained by forming under tension of casting wax pattern on a metal tooth. Owing to the shrinkage of casting wax used (Taggart's) this tension is released when the pattern is chilled and removed from the metal tooth model, causing the two ends of the pattern to draw together slightly. This, with a further contraction of the casting metal, cast in a cool mould, causes the finished clasp to grasp tighter at its ends than at any other point in its circumference. This grip is highly desirable because of the following facts:

Masticating force applied to a clasp type removable bridge tends to move it principally in three directions—gingivally, mesially and distally. Three inclined planes are brought into play in each clasp—one at the occlusal rest and two at the ends of the clasp arms. The tendency of the bridge to act as a wedge is slightly increased by the inclined planes of the occlusial rests, and these forces must be correctly counterbalanced by a strong resistant grip at the *ends* of the clasp arms or the bridge will fail in time, due to the opening and loosening of the clasps or the shifting mesially and distally of the abutment teeth. Comparatively slight gripping power is required to retain the bridge against all ordinary efforts of mastication, but great strength is required in the clasp arms to keep the abutment teeth from migrating and consequent loosening, and to limit the lateral motion of the piece. Theoretically, the use of the round wire clasp gives the greatest insurance against future disintegration of the abutment teeth, but unfortunately the round wire clasp has not the forenamed properties of the cast clasp. If the round wire clasp had succeeded in cases where the cast clasp has succeeded, the cast clasp would have no excuse for existence. The writer is frankly willing to admit that he has, for twenty years, always used the round wire clasp wherever he could, but that he can not now substitute it for the cast clasp where the cast clasp is indicated. He also has had occasion to note, since the cast clasp became popular, its use in many cases of prosthesis where the use of wrought clasps of either wire or sheet clasp metal would have been much better.

The successful prosthodontist must invariably be the one with many methods at his command. No one type of appliance or attachment will fit every case. Depending upon his wise selection and proper application of the many forms of attachments now in use, as well as upon his technical ability, will his work succeed or fail? To those of the profession who, seeing the value and popularity of the cast clasp, have hastened to put out a technic of their own, I have this to say: It is promptness of execution that puts the value into any idea. The best idea in the world is valueless until put into working shape and used. The technic of the cast clasp was put into working shape in 1914, and has been in use ever since. Your essayist believes with the late Theodore Roosevelt, that every man owes some of his time to the upbuilding of the profession or business to which he belongs. The host of friends of the cast clasp bridge already in the dental profession is proof positive that the writer's experimental time was not spent in vain.—*Journal National Dental Assn.*

